# Antibody epitope repertoire analysis enables rapid antigen discovery and multiplex serology

**DOI:** 10.1038/s41598-020-62256-9

**Published:** 2020-03-24

**Authors:** Kathy Kamath, Jack Reifert, Timothy Johnston, Cameron Gable, Robert J. Pantazes, Hilda N. Rivera, Isabel McAuliffe, Sukwan Handali, Patrick S. Daugherty

**Affiliations:** 1 Serimmune Inc., 150 Castilian Dr., Goleta, CA 93117 USA; 2 Present Address: Department of Chemical Engineering, Auburn University, Auburn, AL 36849-5127 USA; 3 Centers for Disease Control (CDC)- Division of Parasitic Disease and Malaria, 1600 Clifton Road, MS D-64, Atlanta, GA 30329-4027 USA

**Keywords:** Antimicrobial responses, Adaptive immunity, Humoral immunity, Biotechnology, Applied immunology

## Abstract

The detection of pathogen-specific antibodies remains a cornerstone of clinical diagnostics. Yet, many test exhibit undesirable performance or are completely lacking. Given this, we developed serum epitope repertoire analysis (SERA), a method to rapidly discover conserved, pathogen-specific antigens and their epitopes, and applied it to develop an assay for Chagas disease caused by the protozoan parasite *Trypanosoma cruzi*. Antibody binding peptide motifs were identified from 28 Chagas repertoires using a bacterial display random 12-mer peptide library and next-generation sequencing (NGS). Thirty-three motifs were selected and mapped to candidate Chagas antigens. In a blinded validation set (n = 72), 30/30 Chagas were positive, 30/30 non-Chagas were negative, and 1/12 *Leishmania sp*. was positive. After unblinding, a Leishmania cross-reactive epitope was identified and removed from the panel. The Chagas assay exhibited 100% sensitivity (30/30) and specificity (90/90) in a second blinded validation set including individuals with other parasitic infections. Amongst additional epitope repertoires with unknown Chagas serostatus, assay specificity was 99.8% (998/1000). Thus, the Chagas assay achieved a combined sensitivity and specificity equivalent or superior to diagnostic algorithms that rely on three separate tests to achieve high specificity. NGS-based serology via SERA provides an effective approach to discover antigenic epitopes and develop high performance multiplex serological assays.

## Introduction

The detection of antigen-specific antibodies in human specimens via serology remains an essential and fundamental necessity in laboratory medicine and therapeutic and vaccine development. Antibody serology is used in the diagnosis of hundreds of infectious, allergic, and autoimmune diseases, and new biomarkers and tests continue to expand the utility of serology. Nevertheless, antibody serologic tests frequently exhibit suboptimal performance characteristics as measured by clinical sensitivity and specificity, require subjective interpretation^1^, and/or depend upon multi-step algorithms^2,3^. Serologic assays using whole cell material and antigen mixtures can have high false positive rates, leading to unnecessary follow-up tests, inappropriate treatments, or misdiagnosis of patients. Antibody serologic methods typically provide a narrow view of immunity towards a single, or small number of antigens or organisms, while an estimated 1400 pathogens may cause human disease^4^. And, many of these diseases lack effective serology tests altogether. Furthermore, many tests remain organism or antigen-based, even though knowledge of which antigen epitopes are targeted can be critical for inferring infection stage^5^, reactivation^6^, and immune protection^7^.

Despite biomedical need, methods have not emerged to broadly analyze antibody repertoire composition to enable multiplex serology for infection, allergy, or autoimmunity. Multiplex serology is typically limited to several organisms, and to only a small subset of their immunogenic epitopes^8,9^, even though infection or vaccination can give rise to hundreds of distinct antibody species^10^. To expand the breadth and information content of parallel immunoassays, pathogen-proteome derived phage display libraries and planar peptide arrays have been developed. For example, a phage display library of 93,904, 56-mer peptides spanning 208 human virus proteomes was constructed^11,12^. Following immunoprecipitation and DNA sequencing, seropositivity towards each virus was inferred. However, comprehensive proteome tiling for many higher complexity organisms is currently impractical given their larger proteome size. Similarly, the tiling of overlapping peptides from sets of *a priori* known antigens from eight tick-borne pathogens enabled construction of a planar peptide arrays with 170,000 features enabling detection of each infection^2^. In principle, this method could be expanded to include a larger number of pathogens but requires known antigens or epitopes, and does not provide sufficient peptide diversity to mimic diverse structural epitopes. Random peptide arrays of up to 330,000 members have proven effective to detect antibodies towards a range of organisms (i.e., viral, bacterial, fungal)^13^. Yet, they lack the diversity required to effectively mimic arbitrary protein antigens, and thereby detect the corresponding antibodies. Thus, methods to analyze entire antibody repertoires to reveal the spectrum of antigenic epitopes are needed.

To enable epitope resolution analysis of immune responses towards any organism, we applied parallel advancements in peptide display library technology^14^, next-generation sequencing (NGS), and computational discovery algorithms^15^. We applied serum epitope repertoire analysis (SERA) to discover shared, but highly specific immunogenic epitope motifs associated with Chagas disease caused by the protozoan parasite *Trypanosoma cruzi*, and thereby develop a serological assay. Chagas disease is estimated to impact more than 300,000 people in the United States and 8 million in Central and South America^16^. Confirmatory testing currently requires the use of *three* serology tests - two parallel, independent tests, and a third “tie-breaker” test to achieve a specificity of >99%. Thus, a single test with high specificity could streamline confirmatory testing and screening in blood donors and at-risk groups^17^. Our results indicate that NGS-based serology using SERA provides an effective approach to antigen and epitope discovery, and an assay format capable of achieving exceptional diagnostic specificity without multiplexing limitations.

## Results

**Figure 1:**
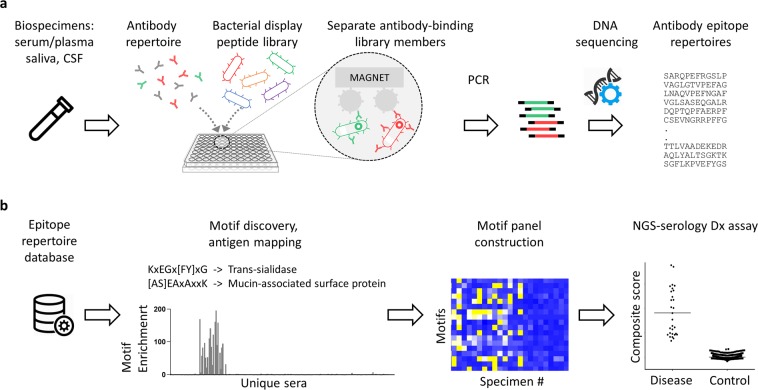
Antibody epitope repertoire analysis (SERA) workflow. (**a**) Each specimen (15 uL) is mixed with a bacterial display random peptide library, (ii) antibody binders are separated using magnetic beads, (iii) a bar-coded amplicon library is prepared from isolated plasmid DNA, and (iv) NGS is performed on the pooled amplicon libraries for ~96 specimens. (**b)** Motifs specific to the cohort of interest are discovered using the IMUNE algorithm and down-selected for specificity, (ii) assembled into a motif panel, and (iii) visualized as a composite score for each specimen.

To demonstrate the utility of SERA in antigen discovery and multiplex serology we applied SERA to discover conserved immunogenic epitopes of IgG antibodies present in sera from individuals with Chagas disease. The SERA workflow consisted of the steps of (i) separation of antibody-binding peptide library members, (ii) preparation and next-generation sequencing (NGS) of amplicon libraries, (iii) computational discovery of disease-specific motifs and motif panel assembly^15^, and (iv) experimental validation of panel performance (Fig. 1). To effectively mimic the diverse linear, structural, and post-translationally modified epitopes from many different organisms, a random peptide library consisting of 10^10^ random 12-mers^15^ displayed on the outer surface of *E. coli* bacteria was used. As a source of diversity, we selected 12-mer random peptides since prior studies of antibody binding epitopes have reported that 95% of linear epitopes span fewer than 12 amino acids^18^. On the other hand, simple structural epitopes (e.g. alpha-helices, beta-hair-pin motifs) can benefit from longer candidate peptides. However, as peptide length grows library quality can deteriorate due to oligonucleotide synthesis errors, or expression and display bias introduced by the peptide display vector. Furthermore, longer peptide sequences (e.g. >15) can contain a larger number of distinct epitopes, thereby increasing opportunities for peptide cross-reactivity with antibodies with divergent specificity. To maintain library stability and diversity during propagation, a tightly regulated expression vector was used for peptide display^14^.

### Discovery of Chagas disease-specific antigen motifs

Biospecimens seropositive for Chagas disease (n = 28) and negative controls (n = 30) were provided by the Centers for Disease Control and Prevention, Division of Parasitic Diseases and Malaria (CDC-DPDM) Reference Lab (Table 1, Supplemental Table S1). Specimens were from males and females, with a mean age of 42+/−17 years, and residing primarily in the southern United States. All disease specimens were seropositive for Chagas disease using the CDC two-test algorithm requiring seropositivity on both the Chagas Antigen ELISA, and a separate immunoblot. One of 28 specimens exhibited discordant ELISA/immunoblot results, and a second-tier IFA test was used to resolve the discordancy. Additional presumed non-Chagas specimens (n = 170) were sourced from commercial vendors.

**Table 1:** Characteristics of specimens used for Chagas motif panel development.

Cohort	Country of origin/travel	Source/Predicate testing	#	Age (yrs) Mean/SD	Gender F/M
Chagas disease discovery	Central and South America	CDC/Chagas EIA, TESA blot, IFA	28	42+/−17	17/10
Chagas control discovery		CDC	30	NA	NA
Specificity controls					
Cysticercosis		CDC	30	NA	NA
Toxocara		CDC	10	NA	NA
Zika virus	Dominican Republic	Bocabiolistics	30	41+/−13	21/9
Healthy donors	USA	Commercial	100	39+/−19	74/26
Blinded validation 1		CDC	72	NA	NA
Chagas (30)					
Negative controls (30)					
Leishmania (12)					
Blinded validation 2		CDC	120	NA	NA
Chagas (30)					
Toxocara (30)					
Toxoplasma gondii (30)					
Cysticercosis (30)					

Each specimen was incubated with the peptide library and antibody binders were separated using Protein A/G-conjugated magnetic beads. After amplifying the selected population through growth, plasmids were prepared and the peptide-encoding regions were amplified via PCR with a unique oligonucleotide bar-code for each specimen. The resulting amplicon libraries were pooled and the DNA sequences of the peptide encoding regions were determined using NGS. On average, ~7 million total sequence reads and ~3 million unique reads were determined for each bar-code and corresponding specimen. The set of unique antibody binding peptides for each specimen, or *epitope repertoire*, was inferred by translation of the DNA sequences into amino acid sequences, and unique sequences were archived for bioinformatic analysis.

**Figure 2:**
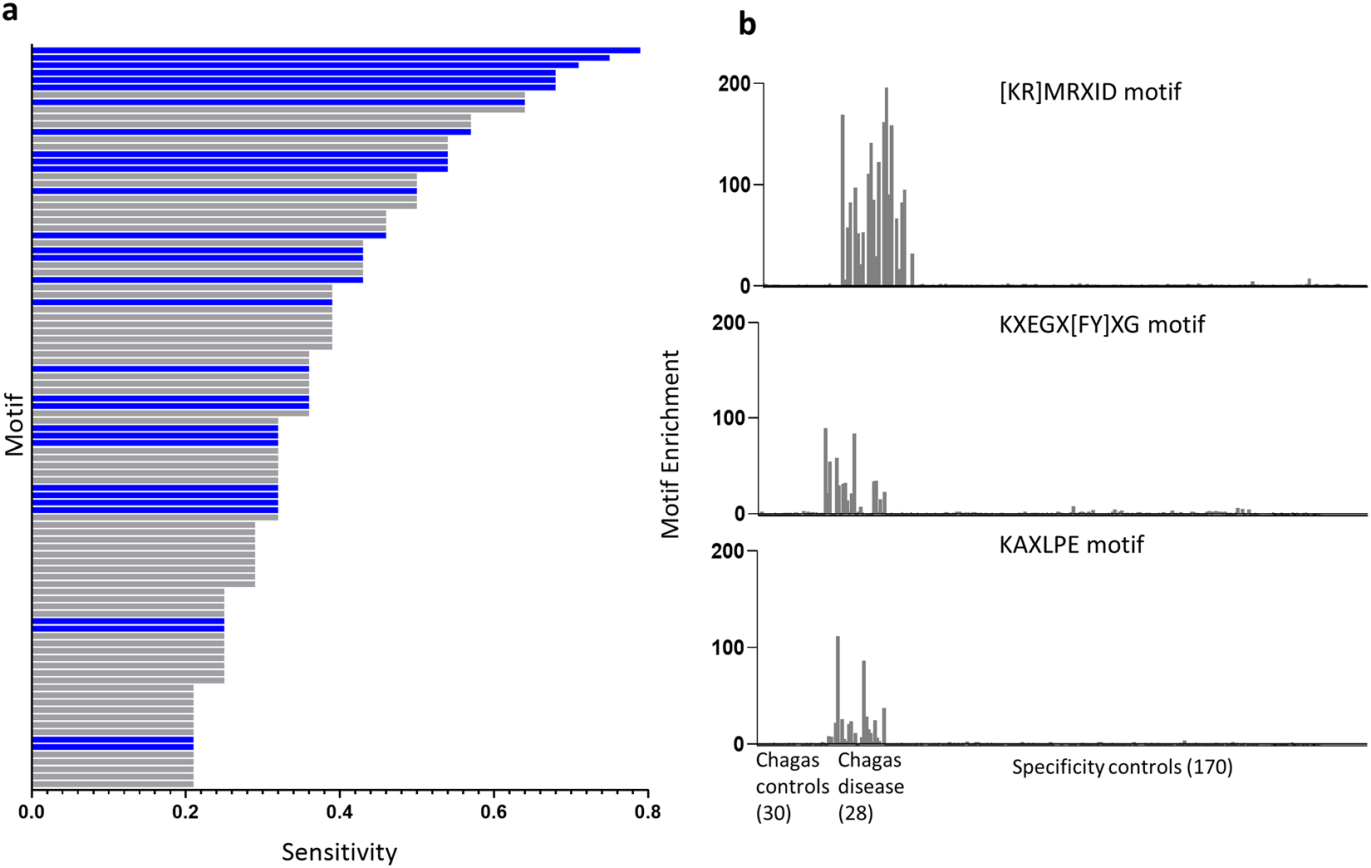
Chagas disease specific motif discovery. (**a**) Sensitivities of the top 100 motifs exhibiting >99% specificity discovered using the IMUNE algorithm. Blue bars – motifs that met criteria (Methods) for inclusion in the panel. Grey bars – motifs that were removed. (**b)** Motif enrichments for three representative motifs in disease (30) and control (200) repertoires used for discovery.

To identify antibody epitope motifs specific to Chagas disease, we applied the IMUNE motif discovery algorithm^15^ (see Methods for parameters) to epitope repertoires from 28 Chagas specimens and 30 non-Chagas sera, yielding 331 candidate motifs (Supplementary Table S2). We then down-selected the full set of IMUNE output motifs by requiring a retained motif to be (i) significantly enriched (≥4 standard deviations from mean of the controls) in at least 15% of Chagas specimens (n = 28) and (ii) absent from control repertoires (i.e., non-significantly enriched (<4 standard deviations from mean of controls) in 99% of control epitope repertoires (n = 200). Downselection yielded 194 motifs with sensitivities ranging from 18–80% (Supplementary Table S2). IMUNE identified overlapping groups of motifs that mapped to the same *T. cruzi* epitopes and occurred in the same subsets of epitope repertoires. For example, [FW]KPWE and EGxKxWE shared three identities, and both occurred within a metacaspase 7-mer epitope EGFKPWE. Motifs mapping to the same epitope were grouped and their equivalence was confirmed by clustering of motif seropositivity (Supplementary Table S3). Thus, IMUNE yielded more than 100 Chagas disease specific motifs (>99% specificity) with varying sensitivity (Fig. 2a). Individual motifs were remarkably specific to Chagas disease, as determined by inspection of their corresponding Enrichment values (# observations/# expected) within the discovery set of 200 epitope repertoires (Fig. 2b).

### Construction of a chagas disease motif panel

**Figure 3:**
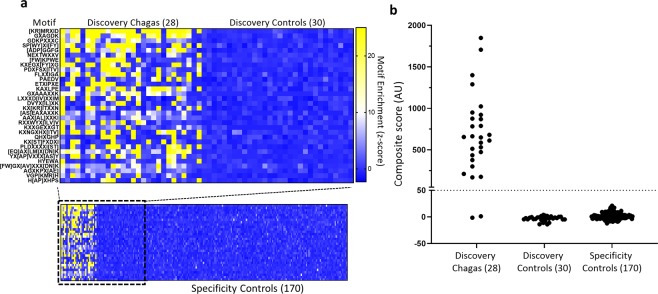
Chagas motif panel v1.0 performance within discovery set. (**a**) Heat map of individual motif z-scores for the 32 motif panel within 28 Discovery Chagas and 30 Discovery Control repertoires, and an additional 170 Specificity Controls. (**b)** Composite (AU) scores for Discovery Chagas and Discovery Control epitope repertoires used in motif discovery.

Motifs within a given epitope group with the highest mean enrichment and sensitivity were selected for inclusion into a diagnostic motif panel. In two cases, two motifs from the same group were included in the panel because their combination increased sensitivity. Chagas-specific motifs exhibited variable enrichments of up to 150-fold and each motif was present in a different subset of disease repertoires (Figs. 2b, 3a). Motif enrichment values were standardized using the mean and standard deviation of enrichments within non-Chagas repertoires. Individual Chagas motif “z-scores” were then summed to obtain a *composite* SERA score for each epitope repertoire. A composite score threshold of 50 readily captured 26/28 serologically defined Chagas specimens with 100% specificity (0/200 controls) (Fig. 3b).

### Motif panel performance in a randomized, blinded validation set

**Figure 4:**
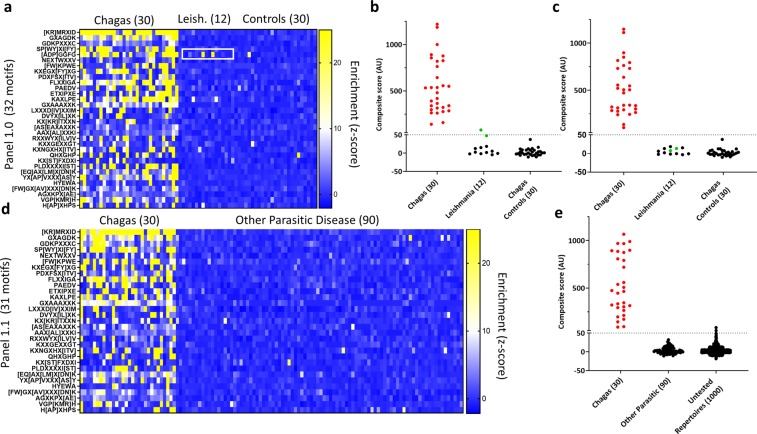
Chagas disease panel performance in randomized and blinded validation cohorts. (**a**) Heat map of motif z-scores after unblinding. Motif [ADP]GGFG was enriched in Leishmania repertoires (boxed region). (**b**) Chagas panel v1.0 exhibited 100% sensitivity and 98% specificity within this cohort of n = 72 sera. Green = Leishmania repertoires positive for the panel motif. (**c**) Removal of the cross-reactive motif (panel v1.1) resulted in 100% sensitivity and specificity. (**d**) Heat map for Chagas panel v1.1 on the second blinded validation cohort. (**e**) Panel v1.1 exhibited 100% sensitivity and 100% specificity within the second blinded cohort (n = 120). Two of 1000 repertoires were positive yielding an estimated lower bound specificity of 99.8%.

To evaluate the performance of Chagas panel v1.0, a blinded and randomized set of 72 biospecimens (Table 1, Supplementary Table S1) was analyzed by SERA and composite scores were calculated (Fig. 4a,b). The set contained 12 Leishmania seropositive specimens, known to cause false positives in Chagas serology tests^19^, due to the relatedness of these organisms. Even so, the panel accurately classified all 30 Chagas as positive, as well as 30 non-Chagas controls and 11/12 Leishmania as negative (Fig. 4a,b). A second Leishmania seropositive epitope repertoire was similarly elevated on Chagas panel v1.0 but classified as negative (Fig. 4b). Inspection of the Leishmania motif enrichments revealed that the motif [ADP]GGFG was enriched in these two Leishmania specimens (Fig. 4a), and present in the proteomes of both *T. cruzi* and *Leishmania*. Removal of this motif from the panel, yielded Chagas panel v1.1 which resulted in a sensitivity and specificity of 100% within the first validation set (Fig. 4c).

To further evaluate the performance of Chagas panel v1.1, we processed an additional 120 blinded and randomized validation sera from the CDC that contained sera from subjects with Chagas disease and three other parasitic diseases (Table 1). Panel scores were again calculated for this set. The panel was 100% sensitive and specific within this second, blinded validation set (Fig. 4d,c). To further investigate the specificity SERA Chagas panel v1.1, 1000 epitope repertoires from individuals with unknown Chagas serostatus were analyzed. Remarkably, just 2/1000 had composite scores above the threshold, thereby yielding an apparent *lower bound* of specificity of 99.8% (Fig. 4e). In summary, Chagas panel v1.1 exhibited 100% sensitivity (60/60) and ≥99.8% specificity within the validation cohorts.

### Identification of candidate antigens from Chagas-specific motifs

SERA Chagas panel v1.1 motifs were queried against the *Trypanasoma cruzi* proteome to identify candidate antigens and their antibody recognition epitopes. Several Chagas-specific motifs occurred as exact matches within established serological antigens, including trans-sialidase, MASP, and CA-2 (Table 2). Some motifs occurred multiple times within repeat regions of their corresponding candidate antigen. For example, there were 11 instances of AGxKPx[AE] within the established antigen trans-sialidase, 51 copies of GxAAAxxK within Surface antigen 2 (CA-2), and 19 copies of Yx[AP]Vxxx[AS]Y within microtubule-associated protein **(**Table 2**)**. A few candidate antigens harboring Chagas-specific motifs, including Metacaspase, and Kinetoplast DNA-associated protein have not been described previously as antigens **(**Table 2**)**. These results demonstrate that motifs frequently contain sufficient information content to identify their corresponding antigens and that SERA identifies *bona fide* pathogen antigens.

**Table 2:** Selected Chagas disease specific motifs and candidate antigens and epitopes.

Motif	Candidate *T. cruzi* Antigen	Candidate Epitope	Sensitivity	Specificity
SP[YW]xI[FY]	Kinetoplast DNA-associated protein	SPYsIF	62%	100%
KAxLPE	Mucin-associated surface protein (MASP)	KAtLPE	63%	98%
KxEGx[FY]xG	Trans-sialidase	KeEGgFtG	43%	99%
ETxIPxE	Trans-sialidase, FL-160-1 epitope	ETeIPsE	42%	98%
PDxFSx[ITV]	Trans-sialidase	PDsFSsT	42%	99%
GxAAAxxK	Surface antigen 2 (CA-2) (51 repeats)	GqAAAgdK	58%	100%
[FW]KPWE & EGxKxWE	Metacaspase	EGFKPWE	45% 32%	96% 99%
[AS]EAxAxxK	Mucin-associated surface protein (MASP) (2 repeats)	AEAaAkaK	27%	99%
KxxGExxGT	Mucin TcMUCII	KstGEatGT	13%	99%
RxxWYx[ILV]V	Mucin-associated surface protein (MASP)	RhqWYaVV	28%	100%
AGxKPx[AE]	Trans-sialidase (11 repeats)	AGpKPaE	33%	100%
GDKPxxxC	Surface protease GP63	GDKPsswC	47%	98%
NExTWxxV	ATPase protein	NEeTWppV	22%	100%
HYEWA	Lanosterol synthase	HYEWA	17%	100%
Yx[AP]Vxxx[AS]Y	Microtubule-associated protein (19 repeats)	YrPVdpsAY	55%	100%

## Discussion

Here we present a general methodology for serum epitope repertoire analysis (SERA) to rapidly discover the immunogenic epitopes within an organism proteome, and to arbitrarily multiplex the detection of epitope-specific antibodies to develop high performance NGS-based serology assays. Within Chagas disease sera, many conserved epitopes were identified as sequence motifs representing the shared amino acid preferences within established and putative candidate *Trypanosoma cruzi* antigens. Measurement of motif enrichments within the set of all Ig-binding peptides for a given specimen (the epitope repertoire) provided a quantitative measure of epitope-specific Ig binding activity that could be multiplexed as desired to detect any number of epitope-specific antibodies. A random peptide library of 10 billion 12-mers provided sufficient diversity to represent all possible 6 amino acid motifs with 99% confidence, and all possible 7-mers with 98% confidence. This diversity is thus about 10,000–100,000-fold greater than that represented by pathogen proteome peptide arrays (e.g., representing a specific pathogen)^11^ or random peptide arrays^13^. SERA differs from reported phage display library immunoprecipitation (PhIP) or VirScan^11^ approaches to antibody repertoire profiling, in that it makes no assumptions about which organisms, strains, or specific epitopes may be targeted by antibodies. Consequently, SERA provides a universal approach that can be applied to analyze the immune response to virtually any biological organism or protein antigen - including pathogens, commensals, allergens, biologics and autoantigens.

The diagnostic accuracy of SERA, measured by combined sensitivity and specificity, was superior to that reported for diagnostics in current use for Chagas disease confirmatory testing. Chagas disease resulting from infection with the protozoan parasite *T. cruzi* may be asymptomatic, and chronic, untreated infection can lead to heart failure and death^20^. Transfusions from asymptomatic, infected donors may transmit the parasite to blood recipients. Consequently, serologic testing for Chagas disease is included in routine blood bank testing of donors. For confirmatory testing and screening, no single serological test achieves adequate specificity, necessitating the use of two or three independent serology tests. Using Chagas disease seropositive specimens, a panel of 31 peptide motifs was identified that exhibited 100% sensitivity (60/60) and 100% specificity (120/120) in independent validation sets. Remarkably, the Chagas SERA assay exhibited 99.8% specificity (998/1000) amongst sera without known Chagas serostatus, which compares favorably to the combined sensitivity and specificity of individual, FDA-cleared tests^17^. The exceptional specificity of SERA could be expected to yield fewer false positive tests, a feature that may be useful in screening and surveillance studies^21^, particularly when multiple tests are performed in parallel.

Six genetic lineages of *T. cruzi* or DTUs (discrete typing units) have been described that infect humans. These DTUs show different geographic distributions and may generate strain specific immune responses^22^. The specimens in our study are from individuals receiving confirmatory testing at the CDC DPDRL, and are of undetermined DTU. However, the 28 specimens used for discovery were from subjects who had lived or travelled in multiple countries where different DTUs are prevalent including Bolivia, Brazil, El Salvador, Mexico, Ecuador, Costa Rica, Nicaragua and Argentina^22^ (Supplementary Table S1). We were able to identify a set of epitope motifs that were conserved among these subjects, and independently validated with a sensitivity of 100% in a randomized, blinded set, demonstrating that the discovered motifs effectively captured the diversity in *T. cruzi* DTUs among the tested population. Nevertheless, our discovery and validation specimens may not represent all DTUs, and thus, expanded validation amongst a larger cohort with known DTU may reveal whether all DTUs are detected with equivalent sensitivity. Additionally, we do not know whether these individuals were in the acute or chronic phase of infection. Testing of subjects in which the phase of infection is known is necessary to determine whether our test has differential sensitivity based on phase of infection.

There are at least two likely reasons for the improved specificity observed with SERA. First, panels are constructed using exclusively specific epitopes within protein antigens and those epitopes that that cross-react can easily be identified and removed. Unlike conventional serology in which whole antigens or organism lysates are often used as detection reagents^3^, SERA excludes a large number of potentially cross-reactive epitopes introduced by using whole protein antigens. Second, computational discovery using epitope repertoires from other infectious diseases enabled optimization of motif combinations to minimize cross-reactivity in non-Chagas sera. We discovered the motif [ADP]GGFG to be enriched in the epitope repertoires from specimens seropositive for either *T. cruzi* or *Leishmania* sp., (an organism known to generate false positive Chagas test results^23^). Identification and removal of the shared epitope did not adversely affect diagnostic panel performance.

SERA enabled rapid discovery and mapping of candidate proteome antigens targeted by antibodies associated with Chagas disease. Many *T. cruzi* strains within seven recognized discrete typing units are known to cause Chagas disease^24^. Using a large library of 10 billion random 12-mer peptides, and computational motif analysis, SERA circumvents the challenge of identifying which particular species and strains should be represented for a given pathogen. Strain level variation, a recurrent problem in infectious disease diagnostics^24^, can be accommodated using motifs constructed from peptides enriched from a random library. Here, Chagas-specific motifs mapped to distinct candidate antigens. Several of these antigens have been reported^25^, while others have not been described previously. Approaches to antigen discovery from biospecimens often require laborious antigen cloning, expression, purification, and reactivity testing^26^. While feasible for small organisms such as viruses, this approach becomes difficult or impractical for organisms with large proteomes including bacteria, protozoans, fungi, and plants.

SERA could potentially be used without major modification within clinical testing laboratories. The primary methodological steps of incubation, magnetic bead pull-down/immuno-precipitation, PCR, and NGS are already performed in infectious disease testing laboratories. And, many laboratories are equipped for bacterial culture. Nevertheless, we anticipate that the assay might be offered initially from a centralized CLIA-certified, CAP-accredited clinical testing laboratory. Longer term, commercial testing labs, and state and federal labs could develop Laboratory-Developed Tests (LDTs) within their existing CLIA labs using peptide library reagent plates, and published motifs and methods. In the authors’ facilities, the assay is routinely performed in 96-well plates, using semi-automated commercial liquid-handling instrumentation, with a two day turn-around time. Finally, the bacterial display peptide library used here could be manufactured economically by conventional fed-batch fermentation, and quality control of the library reagent can be performed using NGS to measure the composition/diversity. We typically sequence 400–500 M library members to ensure maintenance of library diversity and lack of bias towards particular sequences. Thus, we have not identified any barriers to routine use of SERA in a clinical testing lab setting.

Because a single SERA assay can detect an arbitrary number of motifs and thus an effectively unlimited number of pathogens, high-multiplex SERA may be useful for syndromic testing to identify unsuspected underlying causes of disease. Similarly, SERA may be a useful tool for epidemiology studies of associations between organisms, antigens, or epitopes and diseases or syndromes. At present, 15% of cancers globally are associated with bacterial, viral, and parasitic infections and this number will likely rise as new associations are discovered^27^. Similarly, autoimmune and neurodegenerative diseases have long been suspected to be triggered or driven by infections or other environmental factors^27,28^. Identification and removal of environmental drivers of these diseases, as with gluten in Celiac disease, may lead to effective interventions^29^. However, the ability to identify associations has been hampered by the limitations of available serologic approaches, including the requirement to predefine a small set of organisms/strains to investigate, and the inability to differentiate epitope-level immune responses. SERA provides a potential means to overcome these serology platform limitations and provide a broad, high-resolution view of humoral immunity.

## Materials and Methods

### Biospecimens

De-identified specimens along with geographical and associated clinical testing data in this study are described (Table 1, Supplementary Table S1). Specimens from the CDC-DPDM Reference Lab and commercial vendors include the following cohorts: discovery Chagas (n = 28), confirmed negative discovery controls (n = 30), specificity controls (n = 170) from apparently healthy donors (n = 100) and non-Chagas controls from a similar geographical distribution as the Chagas disease cohort (n = 70), blinded validation 1 (n = 72), and blinded validation 2 (n = 120).

### Epitope repertoire capture and NGS

Specimens were incubated with a fully random 12-mer peptide library having estimated diversity of 10^10^, expressed on the surface of *E. coli* in a 96-well, deep-well plate format. Each well contained 10^11^ cells for a 10-fold oversampling of the library. Library preparation, binder selection using magnetic-activated cell sorting (MACS), sample-specific amplicon preparation, and next-generation sequencing (NGS) methods were performed as described (see Supplementary Methods).

### Generation of antibody epitope repertoires

Sequencing data from each sample was processed to generate a non-redundant peptide list of antibody binding epitopes using publicly available software as described^30^. FASTQ DNA sequencing files were deposited into the NCBI Sequence Read Archive (SRA) for public access. Sequence processing occurred in the following steps: (1) FASTQ DNA sequencing data were read, filtered, and trimmed. Filtering removed sequences greater than or less than lengths of 36 bases, sequences with four or more mis-matches or base pair insertions in the regions flanking the peptide region, or sequences with in-frame stop codons. Trimming removed the flanking sequences so that only the library peptide encoding sequences remained. (2) The DNA sequences were translated into peptide sequences of 12 amino acids. (3) Peptide sequences were collapsed into a single sequence if they had nine or more shared identities. Processing thus yielded a unique peptide list (each with a count of one) for each specimen representing an antibody epitope repertoire.

### Chagas disease diagnostic motif panel creation

Following NGS and processing of sequencing data, Chagas specific motifs were discovered using the IMUNE computational algorithm. Motifs were down-selected based on sensitivity, specificity and mean enrichment in disease. The resulting Chagas motif panel v1.0 was then tested against the blinded validation cohort 1 and refined to generate the final diagnostic panel v1.1. The methods used to select Chagas-specific motifs for inclusion in the panel are described in detail below.

### Chagas disease motif discovery using IMUNE

Chagas-specific motifs were discovered by comparing the epitope repertoires from the discovery Chagas cohort (n = 28) with those from discovery controls (n = 30) using IMUNE as previously described^15^. IMUNE identified patterns, defined as three to five amino acids interspersed with undefined residues (“X”) within a 10-amino acid window, that were significantly (Poisson p < 0.0001) enriched in 25% of Chagas samples but not enriched in any discovery control samples (100% specificity). Pattern enrichment was quantified as the ratio of pattern observations in a sample to the expected observations taking into account the amino acid frequencies in the peptide library and total number of sequences for that sample as described^15^. Pattern enrichment significance was evaluated via two statistics: Poisson and standard deviation. The Poisson statistic selected patterns if they were statistically enriched (p < 0.0001) in disease repertoires but not enriched in controls. The standard deviation method identified patterns enriched in disease repertoires relative to those of controls. For the standard deviation method, patterns could be present or enriched in controls but were enriched by >3–4 standard deviations above the controls in disease data sets. Significantly enriched patterns were aligned and scored using a PAM30 substitution matrix and IMUNE^15^, to generate motifs that could include positions where multiple amino acids are observed, indicated by brackets (e.g., [IVL] or [KRQ]. Pattern clustering with IMUNE generated 331 motifs **(**Supplementary Table S2**)**. To identify the most sensitive and specific motifs for inclusion into a Chagas diagnostic panel, motif enrichment values were standardized using,

$${z}_{i}=\frac{x-\mu }{\sigma }$$

where *z*_i_ is the z-score of motif i, *x* is the motif enrichment within a repertoire, *μ* = average enrichment of control cohort, and σ is the standard deviation for motif i in the control cohort. A z-score of ≥ 4 was considered positive. Sensitivity and specificity in 28 Chagas and 200 controls were calculated and motifs with a sensitivity <15% or a specificity of <99% were removed.

### Motif grouping by similarity

After down-selection, the 194 remaining motifs were grouped by amino acid similarity. Motifs were grouped if they shared at least 3 of 5 amino acid identities, resulting in 101 motifs being assigned into 9 groups. Clustering of motif positivity within Chagas epitope repertoires confirmed motif redundancy with each group **(**Supplementary Table S3**)**. The motif within an epitope group with the greatest sensitivity and mean enrichment was included in Chagas panel v1.0. In two cases, two motifs were selected from the same group since their combination improved sensitivity.

The remaining 93 motifs that did not fall into a group were further down-selected based on a specificity of >99.5% (<1/200 controls positive) and/or an average enrichment in the discovery Chagas disease cohort of >5, resulting in 34 retained motifs (Chagas panel v1.0). IMUNE generated motifs, down-selection steps and final selected motifs for panel v1.0 are given in Supplementary Table S2. A “composite score” for all 34 motifs in panel v1.0 was calculated as the sum of z_i_ for each motif. If there was more than one motif in a group, the maximum z-score value amongst the motifs was used to calculate the composite score. Collated enrichment, z-score, and composite score data for Chagas panel v1.0 tested against all samples used in discovery as well as the blinded validation 1 cohort are provided **(**Supplementary Table S4**)**. A composite score value for the motif panel >50 was used as the diagnostic criteria for Chagas disease. After unblinding, a single motif enriched in one Leishmania seropositive specimen was removed, resulting in Chagas panel v1.1. Collated enrichment, z-score, and composite score data for Chagas panel v1.1 tested in the second set of randomized and blinded samples are provided (Supplementary Table S5).

### Identification of candidate antigens

Panel motifs were queried against the *T. cruzi* proteome using ScanProsite (UniProt/TrEMBL) to generate a list of candidate antigens (strain CL Brener – Taxon identifier 353153). Selected candidate antigens representing previously identified and potentially novel Chagas antigens are provided along with sensitivity and specificity values calculated from the combined blinded validation cohorts 1 and 2 of Chagas disease (n = 60) and non-Chagas controls (n = 132) respectively **(**Table 2). Motifs with multiple alignments to a single antigen (repeats) are indicated.

## Supplementary Information

Supplementary table S1.

Supplementary table S2

Supplementary table S3

Supplementary table S4

Supplementary table S5

Supplementary Methods
