# Supplementary material for: Antibody epitope repertoire analysis enables rapid antigen discovery and multiplex serology
Source: Sci Rep. 2020 Mar 24;10:5294. doi: 10.1038/s41598-020-62256-9 (PMC7093460; doi:10.1038/s41598-020-62256-9)
Supplement: Supplementary file 6 — Supplementary Methods [file 41598_2020_62256_MOESM6_ESM.docx]

**Antibody epitope repertoire analysis enables rapid antigen discovery and multiplex serology**

Kathy Kamath^1†^, Jack Reifert^1†^, Timothy Johnston^1^, Cameron Gable^1^, Robert J. Pantazes^1,2^, Hilda N. Rivera^3^, Isabel McAuliffe^3^, Sukwan Handali^3^, Patrick S. Daugherty^1*^

**Supplementary Methods**

**Peptide library preparation.** Five frozen stocks of *Escherichia coli* strain MC1061 [F- araD139 Δ(ara-leu)7696 galE15 galK16 Δ(lac)X74 rpsL (StrR) hsdR2 (rK- mK+) mcrA mcrB1] with surface display vector pB33eCPX (each containing 8×10^10^ cells, 10-fold the diversity of the 12-mer X12 library), were inoculated into five 2 L baffled flasks of 500 mL LB (10 g/L tryptone, 5 g/L yeast extract, and 10 g/L NaCl) supplemented with 34 µg/mL chloramphenicol. The flasks were incubated with vigorous shaking (300 rpm) at 37 °C until the cells grew to OD­_600_ = 1.2. Peptide expression was induced with L-(+)-arabinose at a final concentration of 0.02 % wt/vol for one hour. Cells were collected by centrifugation (3500 rcf for 30 minutes) and resuspended in cold phosphate buffered saline containing 15 % glycerol (PBSG) as a cryoprotectant. The cell suspension was divided into wells of a 96-deep-well plate to yield 8×10^10^ cells per well (10-fold the X12 diversity), frozen, and stored at -80 °C until use in screening.

**Library selection.** The X12 library plate was thawed and specimen serum was added to each well achieving a final dilution of 1:25 immediately followed by thorough mixing by pipetting. Serum antibodies bind to antigen mimic peptides expressed on the surface of the bacteria during a one-hour primary incubation carried out at 4 °C with constant orbital shaking (800 rpm). The cells were then collected by centrifugation (3500 rcf) for 7 minutes, the supernatant was removed, and the cell pellets were washed by resuspending fully in 750 uL PBS + 0.0 5% Tween-20 (PBST). The washed cells were again collected by centrifugation (3500 rcf) for 5 minutes and the PBST supernatant was removed. Cell pellets were resuspended in 750 uL PBS and mixed thoroughly with 50 µL Protein A/G Sera-Mag SpeedBeads (GE Life Sciences, 17152104010350) achieving a final concentration of 6.25 % the beads’ stock concentration. The plate was incubated for one hour at 4°C with orbital shaking (800 rpm). Bead-bound cells were captured in the plate using a Magnum FLX 96-ring magnet (Alpaqua, A000400) for 15 minutes until all beads were separated. Unbound cells in the supernatant were removed by gentle pipetting so as not to disturb the beads, leaving only those cells bound to A/G beads. To exclude residual unbound cells and wash non-antibody interactions of cells to beads, the beads were washed by removing from the magnet, resuspending in 750 uL PBST, and then returning to the magnet for 15 minutes. The supernatant was removed by gentle pipetting after the beads were securely magnetized. After repeating this bead washing 5x, and washed beads and cells were resuspended in 750uL LB with 34µg/mL chloramphenicol and 0.2% wt/vol glucose directly in the 96-deep-well plate and grown overnight with shaking (300 rpm) at 37°C.

**Generation of selected peptide amplicon libraries for each patient.** After growth, cells were collected by centrifugation (3500rcf for 10 minutes) and the supernatant was discarded. Plasmids were isolated in 96-well format using the Montage Plasmid Miniprep_HTS_ Kit (MilliPore, LSKP09604) on a Multiscreen_HTS_ Vacuum Manifold (MilliPore, MSVMHTS00) following the “Plasmid DNA—Full Lysate” protocol in the product literature. NGS ready amplicon preparation was achieved through two rounds of PCR. The first round amplified the variable “X12” peptide region of the plasmid DNA. The second round of PCR barcodes each patient amplicon library with sample-specific indexing primers for data demultiplexing after sequencing. KAPA HiFi HotStart ReadyMix (KAPA Biosystems, KK2612) was used as the polymerase master mix for both PCR steps. Plasmids (2.5 µL/well) were used as template for a first round PCR with 12.5 µL of KAPA ReadyMix and 5uL each of 1uM forward and reverse primers. The primers (Integrated DNA Technologies) contain annealing regions that flank the X12 sequence (indicated in bold) and adapter regions specific to the Illumina index primers used in the second round PCR.

Forward primer: TCGTCGGCAGCGTCAGATGTGTATAAGAGACAGVBHDV**CCAGTCTGGCCAGGG**

Reverse primer: GTCTCGTGGGCTCGGAGATGTGTATAAGAGACAG**GTGATGCCGTAGTACTGG**

A series of five degenerate bases in the forward primer, VBHDV (following IUPAC codes), provide base diversity for the first five reads of the sequencing on the NextSeq platform. These five base pairs were designed to be non-complementary to the template to avoid bias during primer annealing. To reduce non-specific products, a touchdown PCR protocol was used with an initial annealing temperature of 72 °C with a decrease of 0.5 °C per cycle for 14 cycles, followed by 10 cycles with annealing at 6 5°C. The 25 µL primary PCR product was purified using 30 µL Mag-Bind TotalPure NGS Beads (Omega Bio-Tek, M1378-02) according to the manufacturer’s protocol. The second round PCR (8 cycles, 70 °C annealing temperature) was performed using Nextera XT index primers (Illumina, FC-131-2001) which introduce sample specific 8 base pair indices on the 5’ and 3’ termini of the amplicon for sample demultiplexing. PCR 1 product (5 µL) was used as a template for the second PCR with 5uL each of forward and reverse indexing primers, 10 µL Tris-HCl and 25 µL of KAPA ReadyMix. The PCR product (50 µL) was cleaned up with 56 µL Omega Mag-Bind TotalPure NGS Beads per reaction.

**DNA quantitation, normalization, and pooling.** A 96-well quantitation was performed using the Qubit dsDNA High Sensitivity assay (Invitrogen, Q32851) adapted for a microplate fluorimeter (Tecan SPECTRAFlour Plus) measuring fluorescence excitation at 485 nm and emission at 535 nm. Positive (100 ng) and negative (0 ng) controls, included with the Qubit kit, were added to the plate as standards along with 2 µL of each PCR product diluted 1:100 in Qbit reagent for quantitation. The fluorescence data were used to calculate DNA concentration in each well based on the kit standards. To normalize the DNA and achieve equal loading of each patient sample on NGS, the DNA in each well was diluted with Tris HCl (pH 8.5, 10 mM) to 4 nM and an equal volume from each well was pooled in a Lo-Bind DNA tube for sequencing.

**NGS of amplicon pool.** The sample pool was prepared for sequencing according to specifications of the Illumina NextSeq 500. Due to the low diversity in the adapter regions of the amplicon after the first five bases, PhiX Run Control (Illumina, FC-110-3001) was included at 40 % of the final DNA pool. The pool was sequenced using a High Output v2, 75 cycle kit (Illumina, FC-404-2005).
